# Predicting the potential distribution of *Amblyomma americanum* (Acari: Ixodidae) infestation in New Zealand, using maximum entropy-based ecological niche modelling

**DOI:** 10.1007/s10493-019-00460-7

**Published:** 2020-01-21

**Authors:** R. K. Raghavan, A. C. G. Heath, K. E. Lawrence, R. R. Ganta, A. T. Peterson, W. E. Pomroy

**Affiliations:** 1 Department of Diagnostic Medicine/Pathobiology, Kansas State University, Manhattan, KS, USA; 2 School of Veterinary Science, Massey University, Palmerston North, New Zealand; 3 Agresearch Ltd., C/O Hopkirk Research Institute, Private Bag 11008, Palmerston North 4442, New Zealand; 4 Department of Ecology, The University of Kansas, Lawrence, KS, USA

**Keywords:** Ecological niche modelling, Tick, *Amblyomma americanum*, New Zealand, MaxEnt

## Abstract

Although currently exotic to New Zealand, the potential geographic distribution of *Amblyomma americanum* (L.), the lone star tick, was modelled using maximum entropy (MaxEnt). The MaxEnt model was calibrated across the native range of *A. americanum* in North America using present-day climatic conditions and occurrence data from museum collections. The resulting model was then projected onto New Zealand using both present-day and future climates modelled under two greenhouse gas emission scenarios, representative concentration pathways (RCP) 4.5 (low) and RCP 8.5 (high). Three sets of WorldClim bioclimatic variables were chosen using the jackknife method and tested in MaxEnt using different combinations of model feature class functions and regularization multiplier values. The preferred model was selected based on partial receiver operating characteristic tests, the omission rate and the lowest Akaike information criterion. The final model had four bioclimatic variables, Annual Mean Temperature (BIO_1_), Annual Precipitation (BIO_12_), Precipitation Seasonality (BIO_15_) and Precipitation of Driest Quarter (BIO_17_), and the projected New Zealand distribution was broadly similar to that of *Haemaphysalis longicornis* Neumann, New Zealand’s only livestock tick, but with a more extensive predicted suitability. The climate change predictions for the year 2050 under both low and high RCP scenarios projected only moderate increases in habitat suitability along the mountain valleys in the South Island. In conclusion, this analysis shows that given the opportunity and license *A. americanum* could and would successfully establish in New Zealand and could provide another vector for theileriosis organisms.

## Introduction

New Zealand’s relative geographic isolation has not prevented the self- or assisted introduction of many exotic species of plants and animals (McGlone 2006; Williams and Cameron 2006). In fact, deliberate introductions of birds and mammals were common in the late eighteenth and nineteenth centuries (Thomson 1922) before the concepts of extinction, competitive displacement and reduced biodiversity were fully realised.

For a nation that relies heavily on agriculture for economic stability, any organism that threatens productivity is an anathema. Furthermore, with tourism recently edging out dairy as New Zealand’s biggest export earner, it is in the best interests of the country to also promote itself as safe for visitors. This requires that potential human exposure to parasites and disease organisms is also kept to a minimum.

In an effort to maintain barriers against unwanted organisms, biosecurity measures are in place such that incoming passengers are screened for unwanted materials that might introduce exotic diseases and pests. A proportion of aircraft, ships and shipping containers is also examined and fumigated if necessary. Surveillance measures annually intercept many potential threats at the border but only a few organisms have breached it. Mosquitoes, fleas, parasitic mites and ticks are examples of medical and veterinary arthropod pests that are regularly encountered, although the only livestock tick in New Zealand, *Haemaphysalis longicornis* Neumann, invaded and established long before any comprehensive quarantine measures were in place (Myers 1924). Of considerable contemporary interest is the finding of *H. longicornis* in north-eastern USA (Rainey et al. 2018) showing that it and many other species of ticks (Burridge 2011) are still threatening invasion around the globe.

The following tick species are listed as notifiable under the New Zealand Biosecurity (Notifiable organisms) order 2016, *Amblyomma* spp., *Dermacentor* spp., *Rhipicephalus* spp., *Boophilus* spp. and *Ixodes* spp. (exotic). In recent years at least 24 species of ticks have been intercepted in New Zealand (Heath 2013), almost equally divided between humans and companion animals (Heath and Hardwick 2011) as vehicles of entry. Brown dog tick [*Rhipicephalus sanguineus* (Latreille)] is a regular introduction to New Zealand from around the world, but especially from Australia, together with the paralysis tick (*Ixodes holocyclus* Neumann), although occasional specimens of other tick species from most parts of the planet have been intercepted (Heath 2013) including North America. The brown dog-tick has established temporarily in New Zealand on a few occasions (Heath et al. 1980; Bingham 2011), going through at least one cycle, but eradication measures have, as far as is known, been completely successful against such incursions.

Potentially, for an exotic tick to establish, a single, invasive ovigerous female would be sufficient, providing suitable hosts and a favourable bioclimatic match were available near the point of entry. Alternatively a mix of all stages in large numbers, or individual adults of both sexes should provide greater certainty of establishment, again as long as host and microclimatic needs are met. A means of dispersal beyond the initial entry point is necessary to enhance the invasion potential. New Zealand has a wide range of avian and mammalian potential tick hosts, with a smaller number of reptiles. Some of the mammalian species are highly mobile and have larger ranges than others (Heath 2016), further aiding dispersal from an entry point. As an illustration of potential host availability, New Zealand has 27 species of widespread terrestrial mammals (King 2005), within a land area of 268,021 km^2^. The USA by comparison has 490 species of terrestrial mammals (Anon 2019) over a land area of 9,834,000 km^2^. Thus, potential host density comprises one species for each 20,069 km^2^ in the USA and one host species for each 9927 km^2^ in New Zealand, almost double the potential USA host density.

The New Zealand climate is warm temperate with a fully humid, warm summer; represented as Cfb in the Köppen–Geiger classification (Maunder 1970) making it suitable for ticks that inhabit similar regions on the planet at relatively high latitudes, excluding desert ecosystems. The Köppen–Geiger classification is a widely used climate classification system in which climates are divided into five main groups (A–E), in which group C is temperate. The second and third letters further sub-divide the climate group based on precipitation and temperature patterns, respectively.

Tick species offering the most serious risk to New Zealand are those with wide host and geographic ranges, and specifically those adapted to cooler or temperate climates and which are vectors of disease agents of livestock, humans and wildlife. Many species fit this category, and Heath (2013) identified 15 of 45 potential invaders in the genera *Amblyomma, Dermacentor*, *Haemaphysalis* and *Ixodes* that would seem to be ideally suited to New Zealand’s suite of hosts and climate factors. However, one species was not considered in that list (because it was not sympatric with *H. longicornis*), but which has come to prominence in recent times is *Amblyomma americanum* (L.), although it has been intercepted only three times at the New Zealand border (Heath and Hardwick 2011; Rawdon 2019). With *H. longicornis* now established in the north-eastern part of the range of *Amb. americanum* (Springer et al. 2015; Rainey et al. 2018) a comparative assessment of mutual environmental suitability is even more cogent.

*Amblyomma americanum* (the lone star tick) is considered one of the most aggressive tick pests in the United States (Cortinas and Spomer 2013; Stafford et al. 2018). It has a wide distribution along the Atlantic coast, from New York to Florida and west into Texas and Oklahoma (Childs and Paddock 2003). All stages of the tick readily feed on people, companion animals, livestock and wildlife. It is particularly abundant among white-tailed deer, the primary host for adult stage ticks, and has been implicated as a vector of *Ehrlichia chaffeensis* (human monocytic ehrlichiosis pathogen) and *E. ewingi* (human ewingii ehrlichiosis pathogen), as well as *Franciscella tularensis* (tularemia pathogen) and Heartland virus (Bunyaviridae: *Phlebovirus*). These all affect humans. The Southern tick-associated rash illness (STARI) of humans is linked with *A. americanum* bites. The tick also harbours feline cytauxzoonosis parasite (*Cytauxzoon felis*) (Farlow et al. 2005; Reichard et al. 2010; Savage et al. 2013; Raghavan et al. 2016) as well as at least one *Theileria* sp. infective for cattle (Chae et al. 1999). Recently lone-star tick bites have also been linked to α-gal allergy, a bizarre, recurrent, life-threatening allergic reaction to red meat (Commins et al. 2011). Extensive studies have shown that *A. amer icanum* is not a vector for Lyme disease pathogen (*Borrelia burgdorferi*) (Stromdahl et al. 2018), however, it may be involved in the transmission of *Rickettsia rickettsii* and *Rickettsia parkeri* (Berrada et al. 2011; Wright et al. 2015).

One effective approach to understand species distribution is through correlative modelling. (Phillips et al. 2006; Estrada-Peña and Venzal 2007) and in recent years maximum entropy (MaxEnt) models have been used successfully to predict tick distributions, habitat suitability and arthropod vectored disease transmission (James et al. 2015; Donaldson et al. 2016; Raghavan et al. 2016, 2019; Lawrence et al. 2016b). Evaluation of the potential for *A. americanum* to establish in New Zealand serves as a proxy for many tick species some of which have been considered in a general sense, based on laboratory and field data (Heath 1979, 1981, 2013).

The aim of this study was to model the potential distribution of *A. americanum*, in New Zealand using maximum entropy-based ecological niche modelling and to examine how this potential distribution of *A. americanum* could alter under two climate change scenarios.

## Materials and methods

To assess the potential distribution of *A. americanum* in New Zealand several candidate ecological niche models were first calibrated across the tick’s current distribution in North America and the best fitting model transferred to New Zealand’s present-day and future climatic conditions.

### Species distribution data

Lone star ticks are widely present in the eastern, south-eastern, and mid-western United States (Childs and Paddock 2003). Species distribution collection data were obtained from the Walter Reed Biosystematics Unit (WRBU), based in the Smithsonian Institution, which included data from the University of Alberta Entomology Collection and the Australian Museum, Sydney. Occurrence data for *A. americanum* in WRBU database includes diverse spatial resolutions. We selected only those records that had GPS coordinates.

The data were checked for positional uncertainty and those records with an uncertainty > 10,000 m were removed. The selected occurrence data points were then rarefied to an inter-occurrence separation of > 50 km, to remove the influence of spatial autocorrelation on model performance (Veloz 2009; Boria et al. 2014). For model construction 50% of the occurrence data points were used for model calibration and 50% for model evaluation i.e. the model was constructed using half the data points and its accuracy evaluated using the other half.

### Environmental data

The 19 bioclimatic variables (BIO_1–19_) available from the WorldClim (version 2.0) data archive (www.worldclim.org) were used for modelling the ecological niche and estimating the geographic distribution of lone star ticks. Bioclimatic variables are summarized raster data (data layers) derived from average monthly temperature and precipitation values for 1970–2000 and are intended to approximate climate dimensions that are meaningful to biological species (Fick and Hijmans 2017). In order to match the uncertainties with occurrence data, climate data at 10-min (~ 17 × ~ 17 km) spatial resolution was used. Four of these data layers which combine precipitation and temperature information into the same layer (Mean Temperature of Wettest Quarter (BIO_8_), Mean Temperature of Driest Quarter (BIO_9_), Precipitation of Warmest Quarter (BIO_18_) and Precipitation of Coldest Quarter (BIO_19_)) were excluded a priori because these data layers have been shown to have spatial artefacts that could affect modelling (Escobar et al. 2014). The relevance of the remaining 15 data layers to the occurrence data was assessed in the model selection step, described in detail below.

### Ecological niche modelling

The machine-learning technique called maximum entropy modelling, MaxEnt (v.3.3.3), (Phillips et al. 2004) was used for estimating the ecological niche of lone star ticks across New Zealand. The modelling approaches used in this study have been discussed and described in detail, both in general terms (Peterson et al. 2011) and for specific disease-relevant systems (Peterson 2014). Parametrization of MaxEnt models have large implications for model outcomes, and one persistent challenge when niche modelling with MaxEnt is choosing appropriate parameter values. In the past, this has been largely done “by art” but more recently quantitative and more robust approaches have been used (Warren et al. 2014; Muscarella et al. 2014). In the present study, many of the recent advances in model selection protocols were utilised, as well as those developed by (Peterson et al. 2018).

Advantages to model performance has been shown when niche models are calibrated across areas that are reasonably accessible to a species due to natural dispersal and other means (Barve et al. 2011; Owens et al. 2013). Therefore, we calibrated our models across an area representing ‘**M**’ within 7 degrees of all occurrences in this study.

Eventually it could be possible for *A. americanum* to establish in the west coast of N. America, however the museum records at UC Berkeley and other west coast museums indicated to us that this species is not present in this region at the present time. Therefore, we did not include these areas for calculating **M**. A summary of the model build follows.

In all, 285 MaxEnt models were built with five different combinations of model feature class functions (i.e. linear; linear and quadratic; linear, quadratic and product; linear, quadratic, product and threshold; and linear, quadratic, product, threshold and hinge), 19 regularization multiplier (RM) values (0.1–1 with intervals of 0.1; 1–6 with intervals of 1; 8–10 with intervals of 2; 10–20 with intervals of 5) and three sets of environmental variables. The RM values determine how snugly the model response fits to the observations in the environmental space.

The three separate sets of bioclimatic variables were selected based on exploration of variable contributions towards the spatial distribution of *A. americanum* via the jackknife procedure (Phillips et al. 2004). Briefly, at each step of the jackknife procedure different models were built with sets of progressively fewer variables. At each step the jackknife procedure removed the least contributing variable(s) for the previous model. The variables included in the final three variable sets were kept as assessed in our model selection procedure. This procedure was aimed at building models that (1) were statistically significant, (2) correctly predicted independent subsets of occurrence data, and (3) adequately described the complexity of the modelled relationship of occurrences to the environmental data (Warren and Seifert 2011; Radosavljevic and Anderson 2014).

Models were selected using three criteria; first they were separated based on partial ROC tests (Peterson et al. 2008), removing non-significant models (*P* > 0.1) from further consideration. Second, the remaining models were filtered by omission rate, and all models with an omission rate > 0.1 (10%) (Phillips et al. 2006) were considered inadequate and were removed. In the final undertaking, the models were sorted by lowest Akaike Information Criterion corrected for small sample sizes (AICc) (Warren and Seifert 2011) values, choosing as final models those within 2 AICc units of the minimum among the significant candidate models. The top model(s) were replicated using the bootstrap function in MaxEnt, 10 replicate data sets were chosen by sampling with replacement, and the median values used as an estimate of the present-day spatial distribution of suitable and unsuitable conditions for lone star ticks across New Zealand. Any uncertainty in present-day model predictions was estimated as the range in the suitability values across model parameterizations.

For predicting the future 2050 spatial distribution of *A. americanum* under the two (RCP) scenarios, the optimal present-day model was transferred to the 2 RCPs and 4 General Circulation Models (GCMs) (see descriptions below). Again, the models were replicated ten times with a bootstrap function and the median output was used for model interpretations.

The Mobility-oriented Parity (MOP) (Owens et al. 2013) was assessed to determine the novelty of climate conditions under the present-day conditions in New Zealand relative to the model calibration area in North America. This procedure was also repeated for the future predictions. The purpose of MOP analysis was to identify areas in New Zealand where strict extrapolation, i.e. model predictions outside the calibration range of at least one of the environmental values found in North America, had occurred. Caution is required when interpreting the likelihood of species presence in areas with higher extrapolative predictions (Alkishe et al. 2017).

### Future climate data

Two greenhouse gas emission scenarios, representing medium to low (Representative Concentration Pathway (RCP) 4.5) and high (RCP 8.5) emissions and climate consequences were considered; this allowed uncertainty in expected climate change to be appraised. The climate change projections were made for the year 2050. For the two RCPs, four general circulation models (GCMs), that simulate future climates, were explored: CSIRO MK3 Climate System Model (CSIRO Atmospheric Research), MIROC 5 (Model for Interdisciplinary Research on Climate, Center for Climate System Research, University of Tokyo), NCAR CCSM4 (National Center for Atmospheric Research, Community Climate System Model-4), and CCCMA CANESM2 (Canadian Center for Climate Modelling and Analysis, Canadian Earth System Model-2). All GCM data were downloaded from the Climate Change, Agriculture and Food Security—Climate Data Portal (CCAFS, 2018), at 30-s resolution.

## Results

**Fig. 1:**
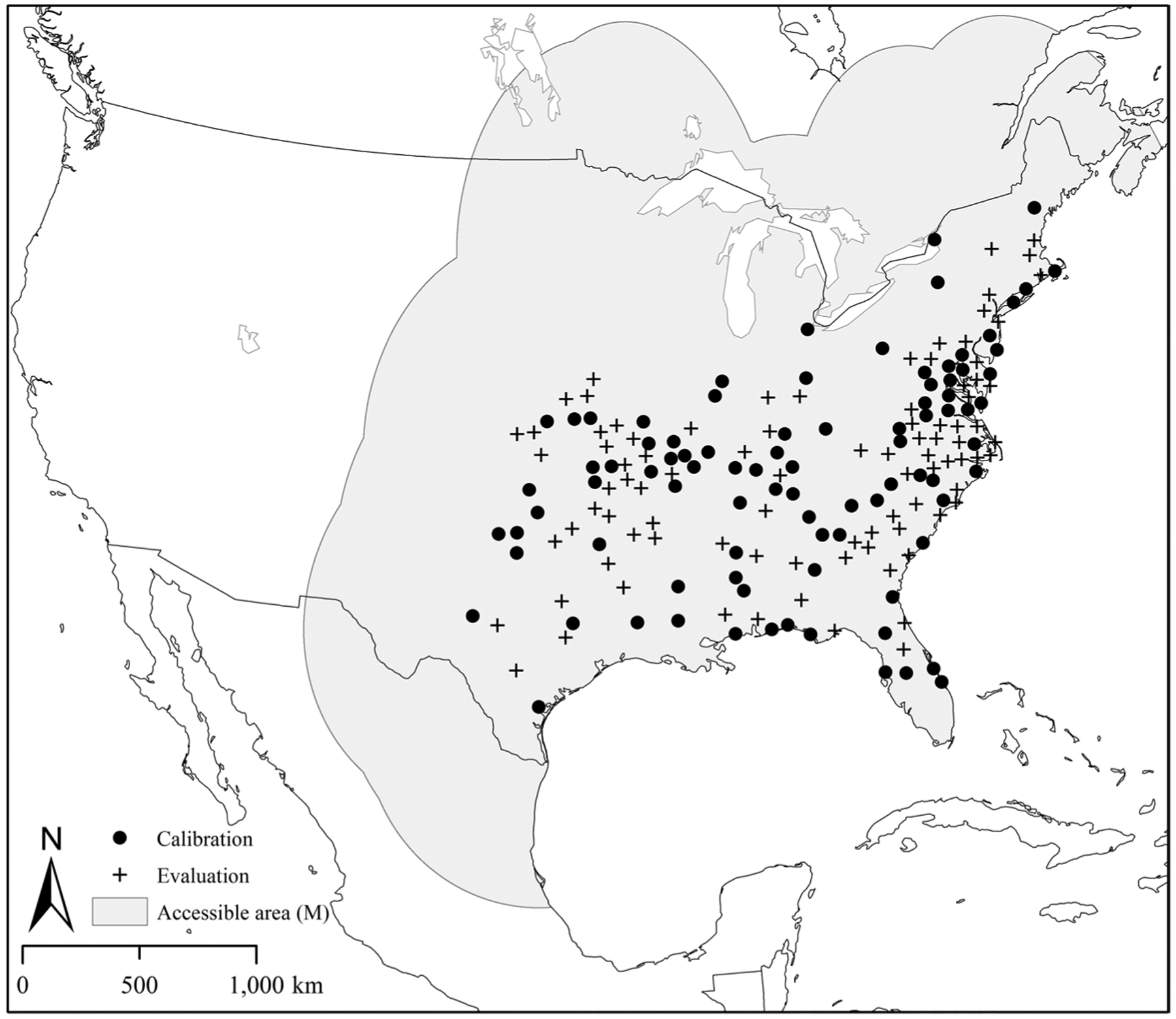
Points representing 185 georeferenced occurrence records of *Amblyomma americanum* ticks used in the analysis and a hypothesized area accessible to this species (**M**) over a period of time. The black dots are the occurrence data points used in the model calibration and the black crosses are the occurrence data points used in the model evaluation

There were 14,831 georeferenced occurrence records available for lone star ticks in the WRBU database. After removing records that did not include uncertainty information and those with uncertainty > 10,000 m resulted in 6492 records. Of these, 33 records were determined to be from outside the known range of lone star ticks. Thirty-one of these records were collected from human subjects reported by the U.S. Army Institute of Public Health, and it is plausible that there were errors in reporting locations where tick exposure occurred. Two other records, one each, submitted by the University of Alberta Entomology Collection and the Australian Museum lacked metadata. The removal of these 33 locations left 6459 occurrence records. After the occurrence data were rarefied to an inter-occurrence separation of > 50 km, there were 185 presence-only locations remaining for analysis. Figure 1 shows the georeferenced occurrence locations used in this study and the areas considered by the authors to be accessible to lone star ticks over time (M).

The evaluation of bioclimatic variable contribution to different models using the jackknife procedure is present in Table 1. The four environmental variables retained in all 4 steps of the jackknife procedure were Annual Mean Temperature (BIO_1_), Annual precipitation (BIO_12_), Precipitation seasonality (BIO_15_) and Precipitation of Driest Quarter (BIO_17_).

In all, a total of 285 models was assessed and all were statistically significant as compared with a null model of random prediction. Six (2.2%) of these significant models met the omission criterion of less than 10%, and, of the significant, low-omission models, the model with the minimum AIC value was selected, which had a regularization parameter value of 2 and the feature class functions were set for linear, quadratic, product, threshold and hinge. This model included the environmental variables in set 3, identified in step 4 of the jackknife procedure, (Table 1). The AIC values of all other models were higher by more than at least 5 units. The jackknife test of the final model showed that Annual precipitation (BIO_12_) had the highest gain when used in isolation and that Annual Mean Temperature (BIO_1_) decreased the gain the most when it was omitted. Meaning that BIO_12_ is the most useful environmental variable by itself and that BIO_1_ has the most information not present in the other three environmental variables.

**Fig. 2:**
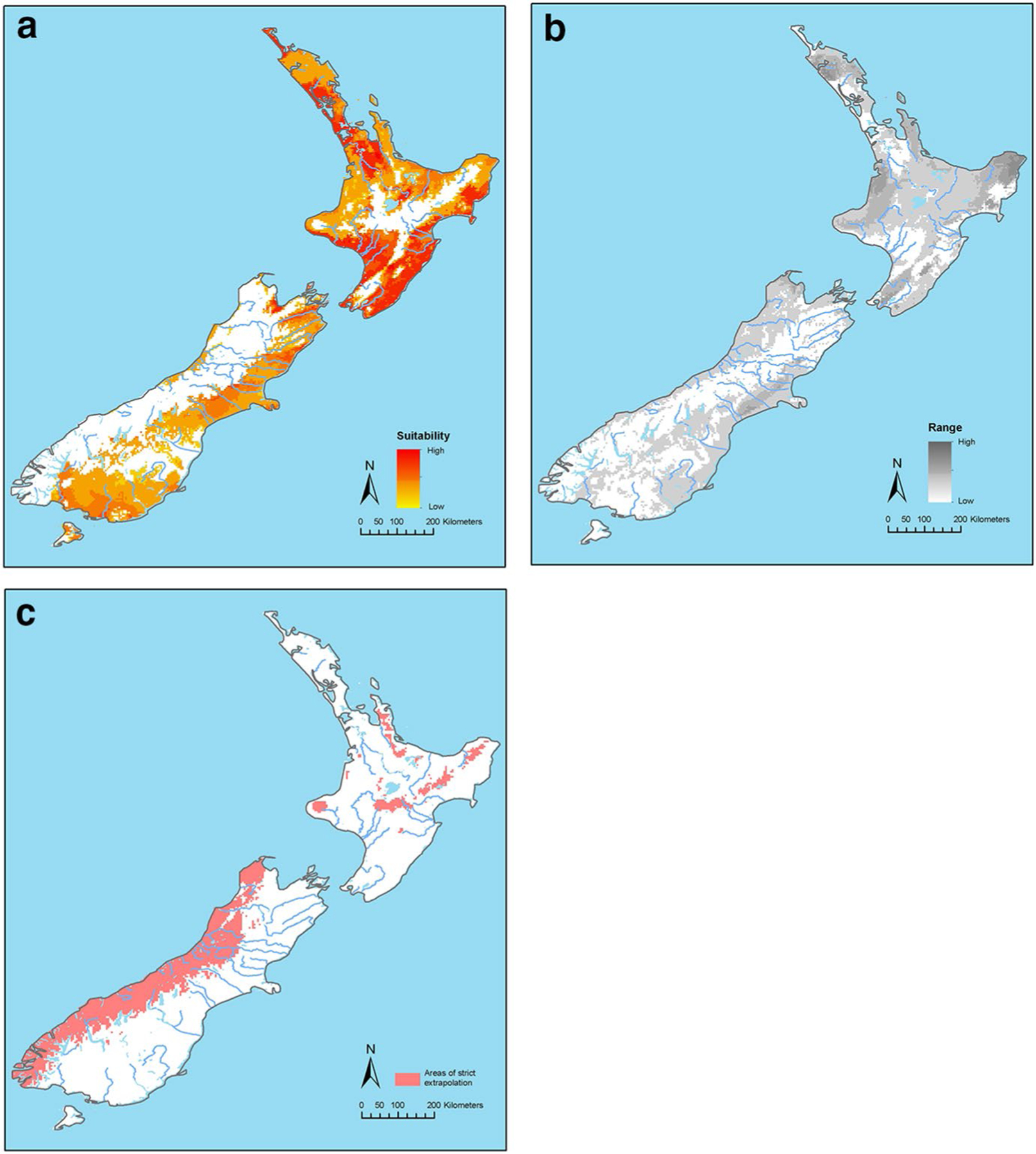
**a** Predicted suitability areas for *Amblyomma americanum* ticks under present-day climatic conditions in New Zealand, as modelled with MaxEnt. White areas are unsuitable and yellow to red areas are increasingly suitable. **b** Uncertainty associated with the predicted suitability areas for *A. americanum* ticks under present-day climatic conditions in New Zealand, as modelled with MaxEnt. The uncertainty increases from white to dark grey. **c** Areas representing strictly extrapolative prediction of suitability for *A. americanum* ticks under present-day climatic conditions in New Zealand. The red areas are extrapolative and predictions for these areas should be regarded with considerable caution (Owens et al. 2013).

The median of the best model, here forward mentioned as present-day model (Fig. 2a), identified areas with different levels of suitability for lone star ticks across New Zealand. Large areas of New Zealand are predicted to be highly suitable. In the North Island, these areas are concentrated predominantly along the south, south-eastern and north central parts of the North Island. However, the other areas in the North Island were also suitable in the medium to low range for the tick. In the South Island, suitable areas in the high to medium range include large areas of the north and eastern half of the island extending to cover large contiguous areas of the south-eastern part of the island. Of note is that much of the mountainous regions and the valleys in both islands are low suitable areas under the present-day climate conditions. The present-day model had large areas with a low uncertainty throughout the predicted distribution range (Fig. 2b). The MOP analysis revealed areas such as the West Coast in the South Island and some mountainous regions in the North Island, for which predictions were strictly based on model extrapolation (Fig. 2c).

**Fig. 3:**
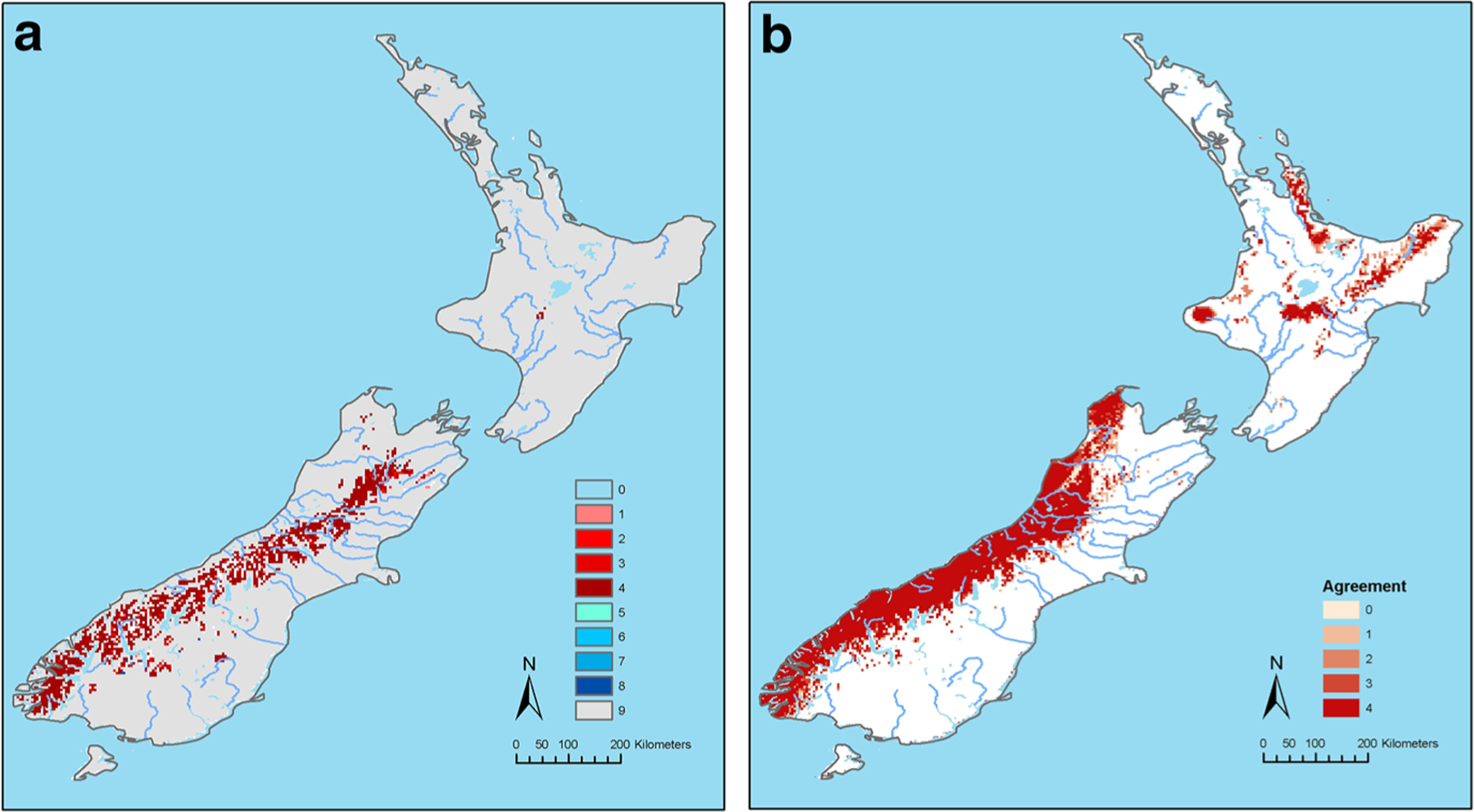
**a** The agreement in predicted suitability of areas for *Amblyomma americanum* ticks between different Global Circulation Models (GCMs) under the Low Emission, Representative Concentration Pathway (RCP) 4.5 Emission Scenario. Where 1 = areas in which one of the four GCMs predicted suitability for *A. americanum* distribution. 2, 3, 4 = two, three and four GCMs predicted suitability, respectively. 5 = areas in which one of the four GCMs predicted loss of territory for *A. americanum* compared to the present-day distribution. 6, 7, 8 = two, three, and four GCMs predicted loss of territory, respectively. 9 = no observed change from predicted current *A. americanum* distribution. **b** Degree of agreement between Global Circulation Models (GCMs) on extrapolative prediction of suitability areas for *A. americanum* ticks under the Low Emission, Representative Concentration Pathway (RCP) 4.5 Scenario. Where 1 = Areas in which the prediction based on one of the four GCMs were strictly extrapolative. 2, 3, 4 = degree of agreement in strict extrapolative areas based on two, three and all four models, respectively

**Fig. 4:**
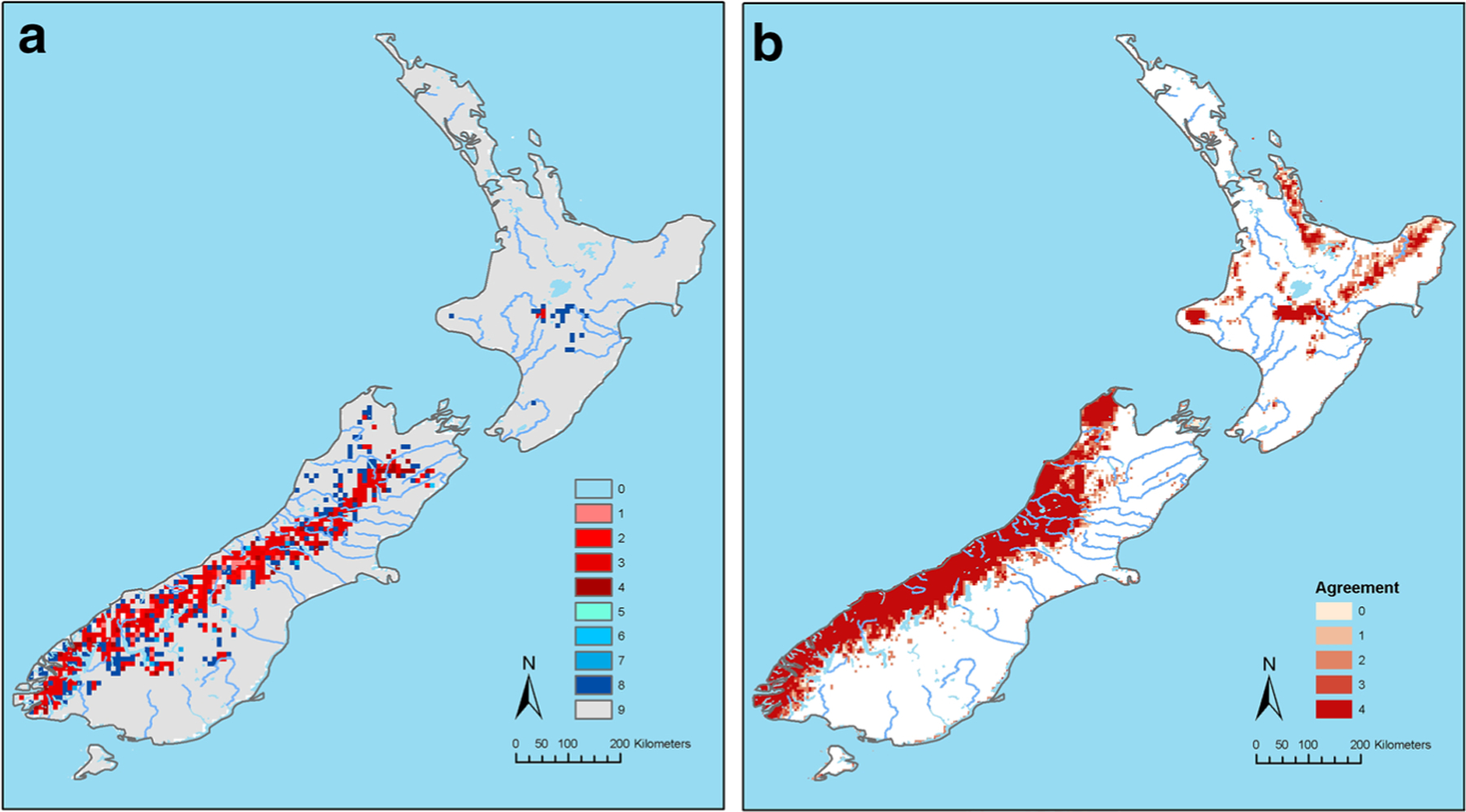
**a** The agreement in predicted suitability of areas for *Amblyomma americanum* ticks between different Global Circulation Models (GCMs) under the High Emission, Representative Concentration Pathway (RCP) 8.5 emission scenario. Where 1 = areas in which one of the four GCMs predicted suitability for *A. americanum* distribution. 2, 3, 4 = two, three and four GCMs predicted suitability, respectively. 5 = areas in which one of the four GCMs predicted loss of territory for *A. americanum* compared to the present-day distribution. 6, 7, 8 = two, three, and four GCMs predicted loss of territory, respectively. 9 = no observed change from predicted current distribution. **b** Degree of agreement between Global Circulation Models (GCMs) on extrapolative prediction of suitability areas for *A. americanum* ticks under the Low Emission, Representative Concentration Pathway (RCP) 4.5 Scenario. Where 1 = Areas in which the prediction based on one of the four GCMs were strictly extrapolative. 2, 3, 4 = degree of agreement in strict extrapolative areas based on two, three and all four models, respectively

The potential for future suitability and distribution of *A. americanum* under both medium to low and high emission scenarios did not change by a large area compared to the potential present-day suitability (Figs. 3a, 4a); but, under both scenarios there were moderate increases in the suitability for this tick species along the mountain valleys in the South Island. Under the high emission scenario, there were some loss of territory compared to the potential present-day distribution (Fig. 4a). The MOP analysis of suitabilities in New Zealand in the future under both emission scenarios (RCP 4.5 and 8.5) compared to the calibration area in North America revealed that predictions for areas predominantly in the mountains and valleys in South Island were strictly extrapolative (Figs. 3b, 4b).

## Discussion

### Predicted distribution

In the present study, the projected distribution for *A. americanum* shown in Fig. 2a closely matches that known for *H. longicornis* in New Zealand, although parts of the South Island that are predicted as suitable for *A. americanum* are slightly more extensive than those currently predicted as a possible extension of the range of *H. longicornis* (Lawrence et al. 2017).

The 4 Bioclim variables included in the final model were Annual Mean Temperature (BIO1), Annual Precipitation (BIO12), Precipitation Seasonality (Coefficient of Variation) (BIO15) and Precipitation of Driest Quarter (BIO17). The annual mean temperature is the average monthly average temperature, the annual precipitation is the sum of all the monthly rainfall, the precipitation seasonality is the ratio of the standard deviation of the monthly rainfall to the average monthly rainfall and precipitation of driest quarter is the total rainfall for the driest consecutive months. The jackknife test identified the two most important environmental variables were Annual Mean Temperature (BIO1) and Annual Precipitation (BIO12).

The largest proportions of the tick’s life cycle, by time, are occupied by the free-living stages which are spent mostly hidden under herbage at ground level. Water exchanges in ticks only occur through the general cuticle surface (Browning 1954), thus the hydration status of the tick will depend on the ambient temperature, rainfall and the atmospheric water pressure. These requirements explain why these two variables are so important.

Within New Zealand, mapping and modelling exercises (Heath 2016; Lawrence et al. 2017) show *H. longicornis* to be widespread in broadly defined, climatically suitable areas, at least when viewed on a large scale. However, a shift of focus reveals a diverse pattern within landscapes, comprising a mosaic of topography (physiography), vegetation and land use types. Within this mix are ecosystems with biotopes that are less suitable for tick survival or proliferation, or have a lower density of hosts than others. For instance, south-facing slopes, short grass, and excessively wet areas are less likely to be a suitable *H. longicornis* habitat. In contrast, warmer north-facing slopes, long but not overly wet pasture, and areas that offer shelter to livestock and wild hosts provide more survival opportunities for ticks (Heath 2016).

These favoured ecotypes are defined by macro and microclimates, and the vegetation and land use patterns they allow, as discussed below.

### Prediction variables

Figure 2b depicts uncertainty associated with the predicted suitability area under present day conditions and, broadly, presents more uncertainty around many areas where *H*. *longicornis* occurs commonly and thrives; i.e., Northland, Coromandel, East Cape, Taranaki and northern Manawatu. The implications from this are that not all of the regions populated by *H. longicornis* would necessarily suit *A. americanum* although this appears to be contradicted by the predicted distribution shown in Fig. 2a.

Figure 2c shows areas representing strictly extrapolative predictions of suitability under present day conditions. These cover all of the West Coast, South Island and a small number of high altitude locations in the North Island. With relevance to this, Springer et al. (2015) found that their projected distribution for *A. americanum* excluded a high-altitude region.

The remaining figures show agreement in predicted suitability of areas between different global circulation models under low emission (Fig. 3a), degree of agreement between GCM on extrapolation prediction of suitability under low emission (Fig. 3b), agreement in predicted suitability of areas between different GCMs under high emission and (4a), and the degree of agreement between GCM on extrapolation prediction of suitability under high emission (Fig. 4b).

The predicted distribution in Fig. 3a covers all of the Southern Alps where there are 16 peaks exceeding 3000 m, and a location near the North Island Volcanic Plateau. The predicted distribution shown in Fig. 4a is similar. The predicted extrapolative distribution shown in Fig. 3b is like that in Fig. 2c, as is that in Fig. 4b, which also has a pattern similar to that shown in Fig. 3b. In summary both climate change models project range expansion of *A. americanum* into the mountainous areas of South Island but with the predictions for the mountains and West Coast also being extrapolative. The predominately mountainous areas are unlikely to be suitable for establishment of *A. americanum* under any but extreme climate-warming scenarios, and even then, vegetation and host density would become only gradually suitable over a long period of time following any change. The lower altitude NW South Island and West Coast middle regions are currently suitable in parts for *H. longicornis* and any change to warmer or wetter climates would only benefit ticks, including *A. americanum*.

Despite mountainous areas in the South Island apparently becoming suitable for *A. americanum* under global warming, long periods of snow cover would still be likely to occur at the highest altitudes and could be a deterrent, although snow is not limiting to the survival of active stages of some species of ticks (e.g. *H. longicornis*). They can survive beneath it, but incubation of eggs is inhibited at temperatures below their developmental threshold, around 12 °C for *H. longicornis*.

Clark (1995), found mean activity thresholds [i.e. no movement of ticks] between 7 and 7.6 °C for adults and 9.6 °C for nymphs of *A. americanum* and mean uncoordinated thresholds [movement but uncoordinated] of 12.3 °C for nymphs and 9.1–10.2 °C for adults.

Spontaneous freezing and direct chilling were not considered significant mortality factors in the field but inoculative freezing: ice nucleation around organic or inorganic entities (Costanzo and Lee 2013) is an important cause of overwintering mortality, following 2 h at − 5 and − 3 °C in direct contact with ice (Burks et al. 1996). *Haemaphysalis longicornis* is freeze susceptible (Yu et al. 2014) and gradual exposure to low temperatures can enhance its cold hardiness, while the possible formation of cryoprotective proteins is suggested, but not proven.

Stewart et al. (1998) found that adults of *A. americanum* did not undergo behavioural diapause when winter-exposed. However, developmental diapause can be photoperiodically induced in fed nymphs of *A. americanum* (Pound and George 1988) and it is of the long day type between 10:14 and 12:12 (light:dark) being critical.

Collectively these data suggest that *A. americanum* would respond to low temperatures in a similar fashion to *H. longicornis*, so would find no shortage of suitable habitats in New Zealand.

### Distribution and climate

The New Zealand climate can be generalised as temperate (Cfb) under the Köppen-Geiger classification (Kottek et al. 2006; Heath 2013; Anon 2018), warm tropical in the north and cool temperate further south. Over much of the USA range of *A. americanum*, there are temperate (Csb) and continental (Dfa) climates with warm, dry summers (Csb) and hot summers with no dry season (Dfa). Although New Zealand is generally Cfb this is considered to be a summation of 18 different climate types, some of which readily match the features of those within the North American range of the lone star tick.

### Bioclimatic preferences

The survival and duration of each stage in the tick life cycle are controlled by physical environmental parameters; the bioclimate. This in turn determines the distribution of ticks. From what is known about *A. americanum* physiology and ecology, much of New Zealand would be suitable. Both *H. longicornis* and *A. americanum* are three-host ticks, with each stage spending considerable amounts of time off the host. All stages of *A. americanum* prefer a moisture-rich environment, and there is a critical humid atmosphere necessary for females to produce eggs that incubate successfully with the suggestion that long periods of drought resulted in a decline in tick populations (Lancaster and McMillan 1955). Nymphs are especially intolerant of dehydration (Yoder and Benoit 2003) but that stage can also take up water, so will thrive in dry environments when (and as long as) water becomes available, as can adults (Sauer and Hair 1971). Moulting (Yoder et al. 2012) and hatching (Patrick and Hair 1979) take place when air is fully or nearly fully saturated and eggs can tolerate submersion for a week.

Wet environments can, however, be a disadvantage because fungal infections could arise and increase permeability, doubling water loss from female ticks (Yoder et al. 2006). This suggests that damp habitats, or times of the year with high rainfall could pose survival problems, at least for females. The West Coast of the South Island has very high annual rainfall 2000 to 3000 mm/year, which probably accounts for the extrapolative predictions, and as such could include habitats less suitable for *A. americanum*.

The climate predictor variable with the best explanatory power for *A. americanum* in the model used by Springer et al. (2015) was mean July (mid-summer) saturation vapour pressure, a time when larvae begin to make their appearance. This has interesting parallels with Heath (2016) where the median March (late summer) average vapour pressure was found to be a good fit with *H. longicornis* distribution, being coincidental with the onset and extent of the seasonal activity period of the larva.

### Vegetation & soil types

Vegetation types in which *A. americanum* is found (McCoy 1971) differ from those occurring in New Zealand which has considerable pasture cover, around 40% of total land area (Anon 2007), with the remainder in exotic (mostly conifer) and native forests. *Amblyomma americanum* prefers secondary growth woodland (Brown et al. 2011), with pine forests not a suitable habitat, and neither was meadow with short pasture exposed to sun and wind (Koch 1984). McCoy (1971) however, found ticks on grassy areas that cattle had access to with all stages found on animals grazing in the shade at the base of a levee.

Larvae prefer vegetation cover whilst adults tend to be found in more open ground that is debris free (Brown et al. 2011; Gilliam et al. 2018). Sites with dense cover and little penetration of sunlight (and high humidity) provided greatest protection compared with sparsely forested sites with little understory and litter. In a separate study, nymphs and males were found to have preferred shaded areas while females favoured sunny (sunlit; exposed) sites (Koch and Burg 2006). It was suggested this increased host finding chances because nymphs and adults move independently of hosts and so utilize much of their habitats which would maximize their chances of coming in contact with a host.

Despite apparent ecosytem differences between NZ and USA, as long as temperature and humidity requirements are met and vegetation cover of an appropriate length and location is available it would appear there is little impediment for *A. americanum* to establish in New Zealand.

### Hosts

The lone star tick has a large host range with McCoy (1971) listing historical records of many mammals and birds, including (relevant in a New Zealand context) cattle, horses, deer, dog and cat for all stages; chicken and turkey, larvae and nymphs; sheep, adults only and mouse, nymphs only. Similar extensive host associations are found with *H. longicornis* (Myers 1924; Heath 2016).

### Seasonality

The collective effects of annual weather variables on ticks are reflected in their seasonal activity patterns as each stage goes through its developmental phases.

The seasonal activity of *A. americanum* in a range of states of the USA has been well defined (e.g., Davidson et al. 1994; Kollars et al. 2000; Childs and Paddock 2003; Bouzek et al. 2013; Cortinas and Spomer 2013; Gilliam et al. 2018) In summary, adult and nymphal lone star ticks are generally most active during April (as early in February in Mississippi for adults; Jackson et al. 1996)) through June, and decline markedly in abundance and activity as summer progresses, with October last month for activity of larvae and nymphs. The early-season activity of adult and nymphal ticks precedes that of larvae (Childs and Paddock 2003) with some larvae possibly overwintering in Georgia. In the more southern states, activity generally begins about a month earlier than in northern states, but goes on for the same length of time independent of state latitude. Ticks are not active during November to January but can be found during the remainder of the year. As soil temperatures increase in spring, egg hatching is encouraged (Patrick and Hair 1979). Soil surface temperature affected frequency of hatching more than did soil moisture, with larval survival shorter in a meadow habitat (Patrick and Hair 1979). High temperature and low humidity extended larval pre-activity (post-hatch movement) out to over 4 weeks in late summer (Patrick and Hair 1979).

Fed larvae, engorged and flat nymphs, unfed adults all overwintered in one study (McCoy 1971), but unfed larvae did not. If these data are extrapolated to a Southern Hemisphere scenario, they show that *A. americanum* would require little if any seasonal adjustment to New Zealand climatic conditions and would be active over most of the year as is *H. longicornis*, and would provide yet another vector for theileriosis organisms that threaten the dairy industry, not just in New Zealand (Lawrence et al. 2016a), but potentially in the USA (Chae et al. 1999; Oakes et al. 2019).

## Conclusion

The lone star tick was not chosen for this modelling exercise because the authors believe there is an imminent risk of its incursion into New Zealand. It was chosen because it is now effectively considered sympatric with *H. longicornis* given the substantial recent spread of this species in eastern USA leading to some overlap in range of both species, and because there exist extensive occurrence data from museum collections in the USA, on which to calibrate an accurate ecological niche model. The model supports the conclusions of Heath (2013) that there are many ticks worldwide that given the opportunity and license could successfully establish in New Zealand.

## Figures and Tables

**Table 1:** Three sets of environmental variables used for three *Amblyomma americanum* MaxEnt calibration models and the four steps of the jackknife procedure used to select the environmental variables in each set

Jackknife step/environmental variable set	Bioclimatic variables used in Maxent calibration model
Step 1	All Bioclim variables^a^
Step 2/Set 1	BIO_1 =_ Annual Mean Temperature BIO_2_ = Mean Diurnal Range (Mean of monthly (max temp—min temp)) BIO_5_ = Max Temperature of Warmest Month BIO_6_ = Min Temperature of Coldest Month BIO_11_ = Mean Temperature of Coldest Quarter BIO_12_ = Annual Precipitation BIO_13_ = Precipitation of Wettest Month BIO_14_ = Precipitation of Driest Month BIO_15_ = Precipitation Seasonality (Coefficient of Variation) BIO_16_ = Precipitation of Wettest Quarter BIO_17_ = Precipitation of Driest Quarter
Step 3/Set 2	BIO_1_ = Annual Mean Temperature BIO_5_ = Max Temperature of Warmest Month BIO_11_ = Mean Temperature of Coldest Quarter BIO_12_ = Annual Precipitation BIO_14_ = Precipitation of Driest Month BIO_15_ = Precipitation Seasonality (Coefficient of Variation) BIO_17_ = Precipitation of Driest Quarter
Step 4/Set 3	BIO_1_ = Annual Mean Temperature BIO_12_ = Annual Precipitation BIO_15_ = Precipitation Seasonality (Coefficient of Variation) BIO_17_ = Precipitation of Driest Quarter

a The following BioClim bioclimatic variables, Mean Temperature of Wettest Quarter (BIO_8_), Mean Temperature of Driest Quarter (BIO_9_), Precipitation of Warmest Quarter (BIO_18_), Precipitation of Driest Quarter (BIO_19_) were a priori excluded from the WorldClim archive because these layers have been shown to have spatial artefacts that could affect niche modelling (Escobar et al. 2014)
